# Pembrolizumab-Induced Lichenoid Dermatitis in a Patient With Metastatic Cancer of Unknown Primary

**DOI:** 10.7759/cureus.13768

**Published:** 2021-03-08

**Authors:** Ashish Sethi, Moses Raj

**Affiliations:** 1 Internal Medicine, Allegheny Health Network, Pittsburgh, USA; 2 Hematology & Oncology, Allegheny Health Network, Pittsburgh, USA

**Keywords:** pembrolizumab, lichenoid drug eruption, supraclavicular lymph node, metastatic cancer of unknown primary, lichenoid dermatitis, psoriasiform rash, cdx2, ck7, lichen planus, immunotherapy-related adverse events

## Abstract

Pembrolizumab is an immune checkpoint inhibitor approved for use in many cancer types such as non-small cell lung cancer (NSCLC), metastatic melanoma, head and neck cancers, hepatocellular carcinoma, and renal cell carcinoma. There are many reported cases of patients on immunotherapy who have discontinued treatment due to the development of immune-related adverse effects (irAE). Recognition of the histopathologic patterns of dermatologic toxicities due to immunotherapy will become increasingly important for ensuring appropriate management and optimal patient care. Here, we present a case of a 72-year-old man with metastatic carcinoma of unknown primary origin treated with pembrolizumab who developed an immune-related cutaneous adverse event (ircAE) in the form of lichenoid dermatitis.

## Introduction

Pembrolizumab is a humanized monoclonal antibody that inhibits the interaction between the programmed death-ligand 1(PD-L1) receptor on T-cells and the PD-L1 and PD-L2 ligands on tumor cells [1]. The interaction of this immunotherapy subtype promotes T-cell reactivation and restores the immune response causing T-cells to attack healthy cells, leading to various autoimmune diseases referred to as immune-related adverse effects (irAE).

Cutaneous toxicities inflict many challenges. A rash is the most common cutaneous toxicity associated with pembrolizumab. Also, it can cause a variety of inflammatory conditions like spongiotic, psoriasiform, and lichenoid dermatitides, mimicking eczema, psoriasis, and lichen planus, respectively. The clinical presentations may be focal or diffuse, including flexural and erythrodermic variants. Pruritus associated with various dermatitides can be severe.

Vitiligo, as an immune-related cutaneous adverse event (ircAE) presents in the form of well-demarcated depigmented macules or patches. Besides contrasting presentation, time to onset varies greatly among such rashes, as vitiligo can appear late after treatment initiation. However, inflammatory dermatoses usually occur within the first one to two cycles into the immunotherapy or pembrolizumab treatment. This requires constant vigilance for signs and symptoms of different cutaneous toxicities. As a result, ircAE has been recognized as a contributing factor to treatment noncompliance, discontinuation, or dose modification. However, with targeted systemic therapies now being available for eczema and psoriasis, correlating the inflammatory patterns of the cutaneous eruptions with the inflammatory patterns that they mimic can help may in more efficacious treatments and fewer drug interruptions and dose modifications. More importantly, it can increase the compliance and efficacy of the pembrolizumab and other immunotherapies.

We report an unusual case of metastasis of unknown primary origin treated with pembrolizumab who developed a widespread lichenoid dermatitis or lichenoid drug eruption.

## Case presentation

A 72-year-old male presented with a pruritic rash on the trunk and extremities of one-week duration. His medical history included metastasis of unknown primary, hypertension, type 2 diabetes mellitus, and hypercholesterolemia.

**Figure 1:**
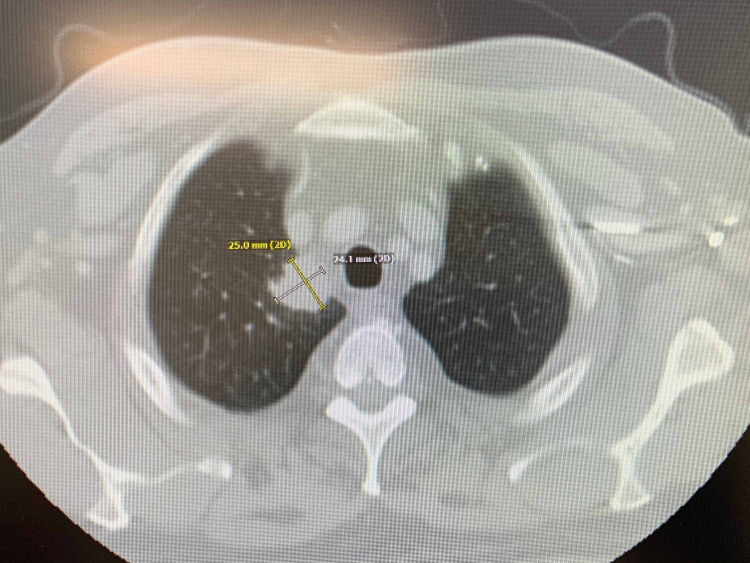
CT chest with contrast Irregular mass concerning for malignancy abutting the right mediastinum within this upper lobe measuring 2.3 x 3 cm. CT: computed tomography

**Figure 2:**
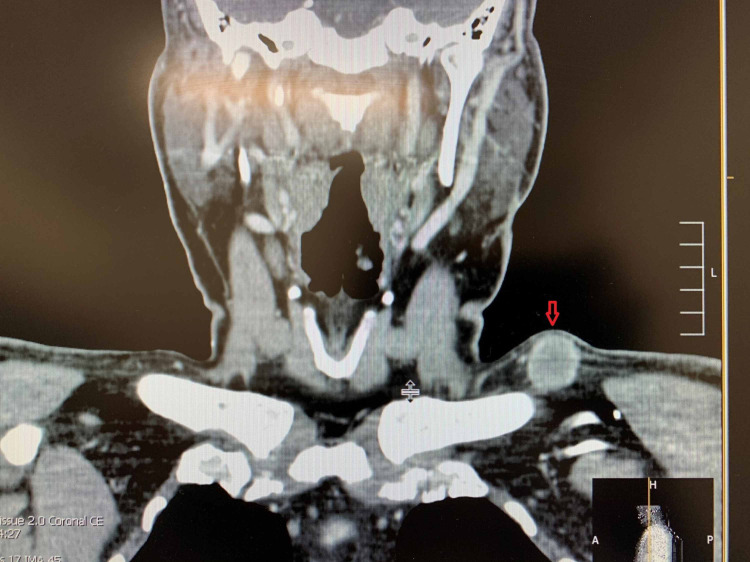
CT neck Red downward arrow: enlarged left supraclavicular lymph node CT: computed tomography

**Figure 3:**
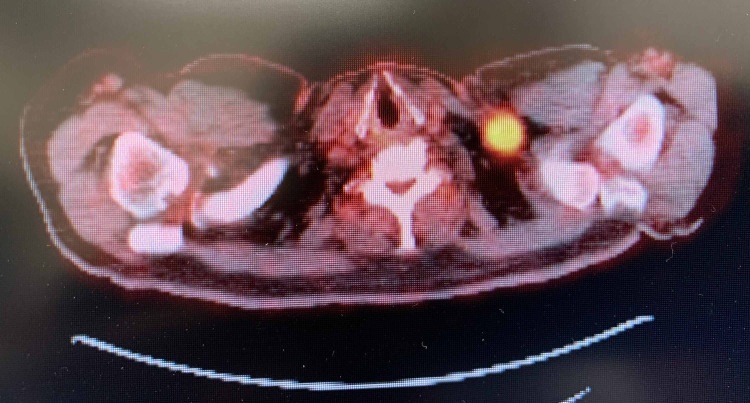
PET scan Enlarged, hypermetabolic lateral left supraclavicular lymph node with maximum SUV of 6.8 SUV: standardized uptake value; PET: positron emission tomography

**Figure 4:**
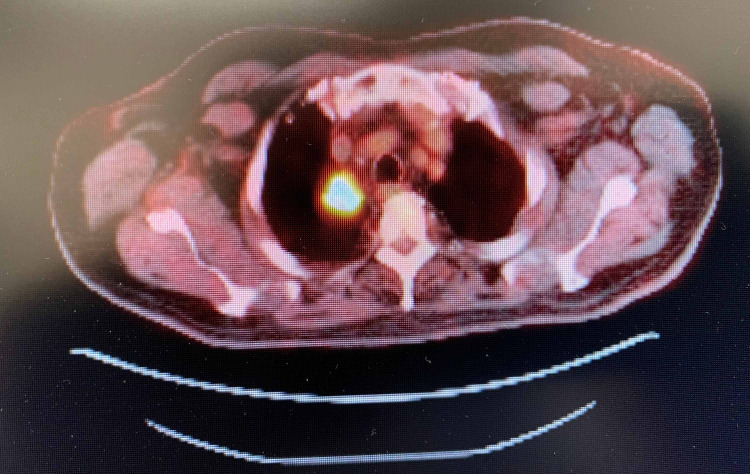
PET scan Hypermetabolic right lung mass abutting the mediastinum, with SUV measuring 14.4 SUV: standardized uptake value; PET: positron emission tomography

**Figure 5:**
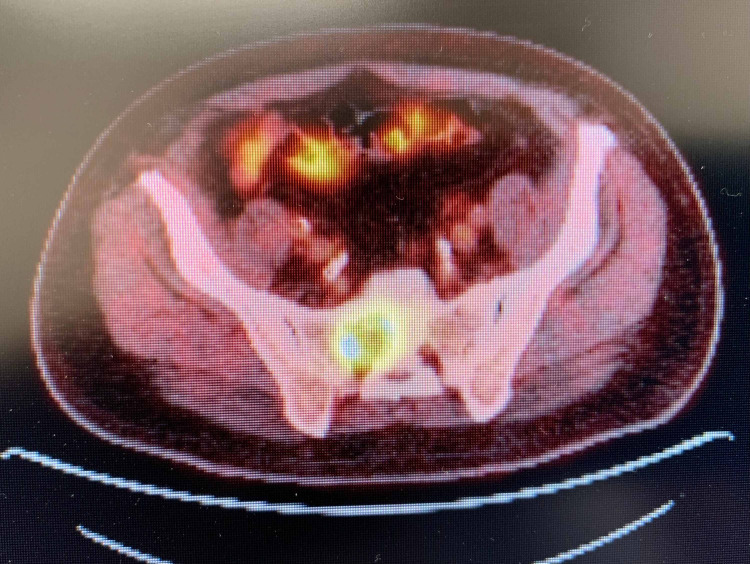
PET scan A large lytic lesion is identified at the right sacrum, with a maximum SUV of 11.4 SUV: standardized uptake value; PET: positron emission tomography

His initial presentation of chronic arthritic pain and lumps in his neck was evaluated by radiological imaging and biopsy. Computed tomography (CT) of the chest and neck revealed a right upper lobe lung mass with mediastinal and supraclavicular lymphadenopathy (Figures 1-2). Subsequently, a fluorodeoxyglucose (FDG)-positron emission tomography (PET) scan illustrated widespread metastatic disease with higher FDG uptake at the right sacrum, lung mass, and left supraclavicular lymph node (SCLN) (Figures 3-5).

**Figure 6:**
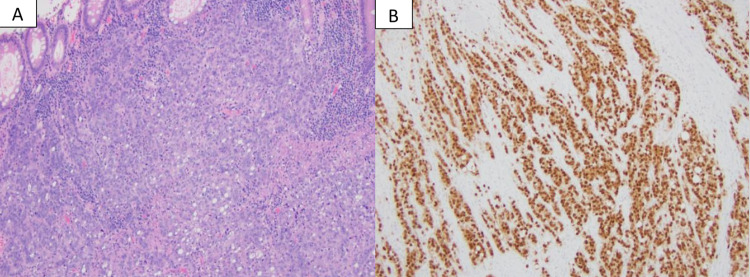
Pathological analysis of the surgical specimen Resection reveals a poorly differentiated adenocarcinoma with a solid and vague glandular growth pattern with many tumor-infiltrating lymphocytes, features of mismatch repair-deficient carcinoma (A, H&E stain). The tumor cells are strongly positive for CDX2 (B, immunostain), supporting a diagnosis of colonic adenocarcinoma. H&E: hematoxylin and eosin Permission was taken from the original publisher; adapted from De Leo et al. [2].

The patient underwent an excisional biopsy of the enlarged left supraclavicular lymph node. Biopsy revealed metastatic, poorly differentiated carcinoma with necrosis and extra-nodal extension. Immunohistochemistry (IHC) performed on the specimen was positive for cytokeratin 7 and CDX2 (Figure 6) and negative for cytokeratin 20, p40, p63, synaptophysin, chromogranin, TTF-1, GATA-3, and PAX8, favoring adenocarcinoma from probably the upper gastrointestinal tract (GIT) as the primary source of origin (Table 1). His liver function tests (LFTs) remained within normal range throughout the clinical course and the carcinoembryonic antigen (CEA) levels were 13 nanograms per milliliter (ng/ml) (Normal CEA: 0-3 ng/ml). He was initially treated with modified FOLFIRINOX based on his clinical presentation and diagnostic evaluation.

**Table 1:** Immunohistochemical stains and analysis CK: cytokeratin; CDX2: caudal type homeobox 2; TTF-1: thyroid transcription factor 1; PAX9: paired box gene 9

Immunohistochemical Stains	Inferences
CK 7 positive and CK 20 negative	Primary is located “above” the diaphragm or from a gynecological origin
CDX2 positive	A gastrointestinal tract, biliary, or pancreatic origin
p40 and p63 negative	Not a squamous cell carcinoma
Synaptophysin and Chromogranin negative	Not a neuroendocrine tumor
TTF- 1 negative	Not a lung adenocarcinoma/small cell primary or a thyroid tumor
GATA 3 negative	Not urothelial or breast primary
PAX 9 negative	Not kidney, thymic, thyroid, and Mullerian as primary

His PDL 1 status was 60% on lymph node biopsy, which was a reasonable choice for starting immunotherapy with pembrolizumab. Molecular studies performed for epidermal growth factor receptor (EGFR), echinoderm microtubule-associated protein-like 4 (EML4), anaplastic lymphoma kinase (ALK) genes, and next-generation sequencing (NGS) were negative. Later, the treatment plan was modified and pembrolizumab was added to the concurrent chemo-radiation regimen with carboplatin and paclitaxel. He also received subsequent radiation therapy to the thoracic spine, left pleural metastasis, right tibia, and thenar eminence of the left hand in view of his disease spectrum and pain at these sites.

**Figure 7:**
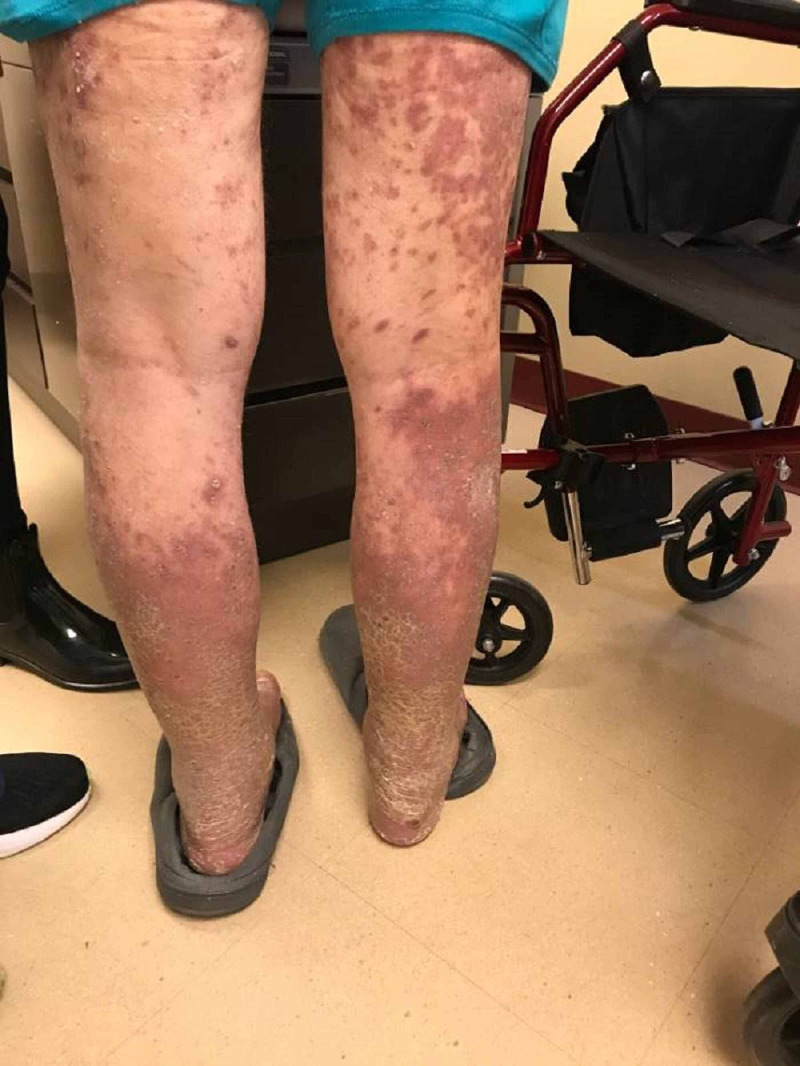
Cutaneous manifestations of lichenoid eruption - lower extremities -Pembrolizumab-induced -Erythematous to violaceous eruption of hyperkeratotic papules and plaques on the legs

**Figure 8:**
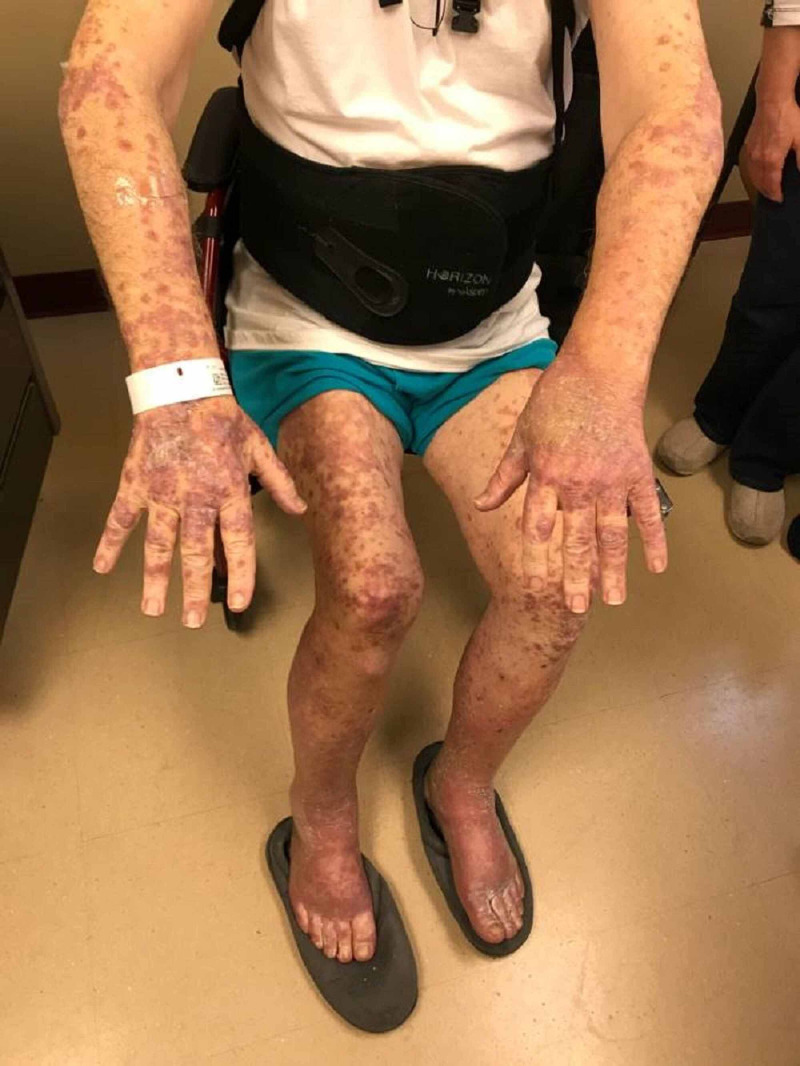
Lichenoid eruption - upper and lower extremities Pembrolizumab-induced

**Figure 9:**
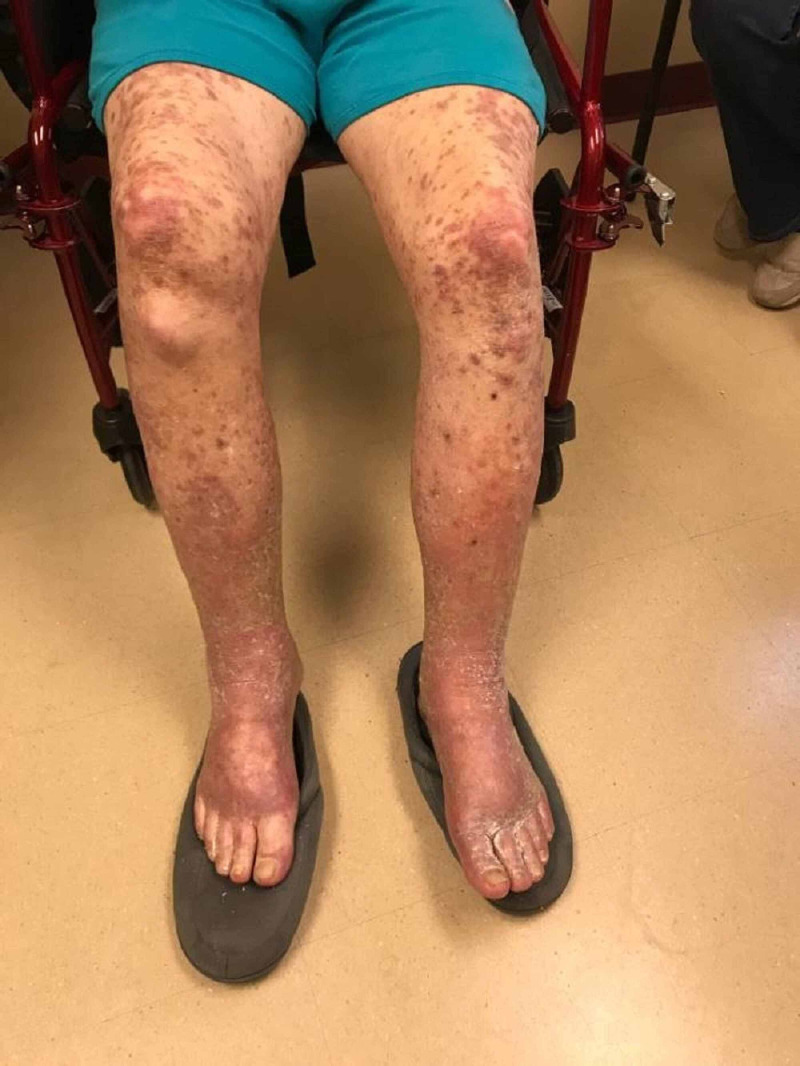
Lichenoid eruption - lower extremities Pembrolizumab-induced

**Figure 10:**
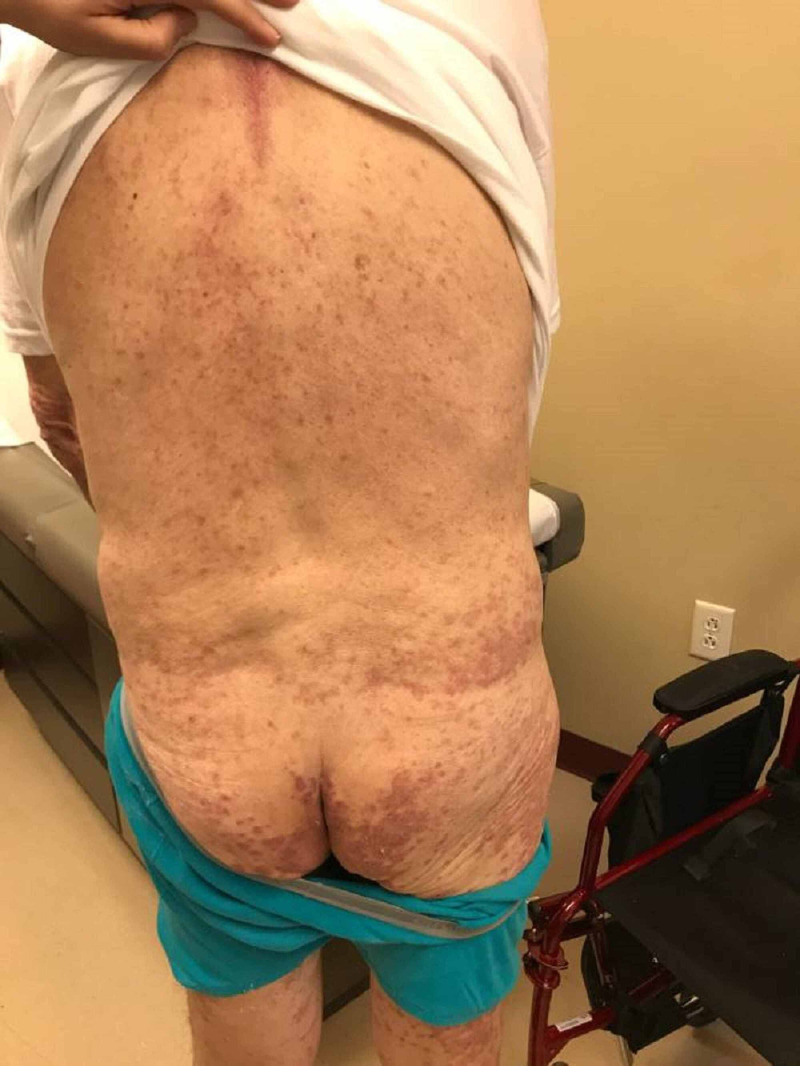
Lichenoid eruption - gluteal region and back Pembrolizumab-induced

**Figure 11:**
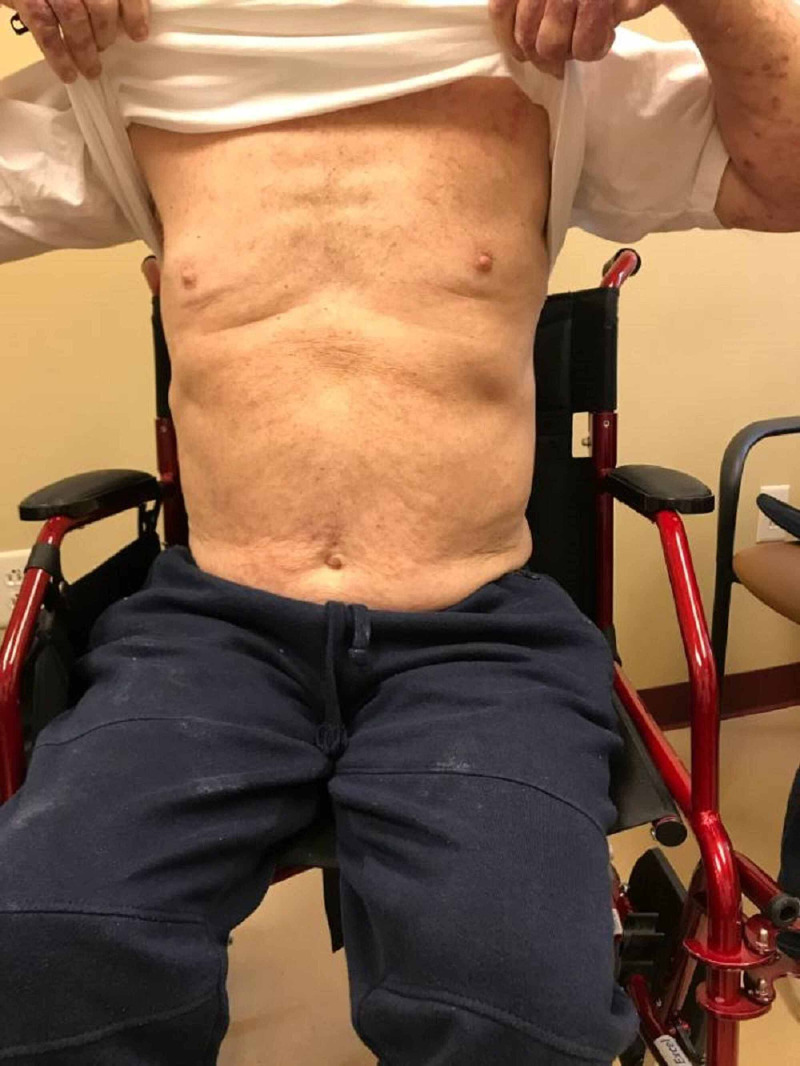
Lichenoid eruption - chest and abdomen Pembrolizumab-induced Faintly erythematous, papules and plaques

After the second cycle of pembrolizumab with a cumulative dose of 400 mg, the patient reported multiple, similar-appearing erythematous, firm papules and plaques, which were pruritic and progressive in nature in the chest, gluteal region, and both upper and lower extremities (Figures 7-11). It was initially diagnosed as psoriasiform drug eruption and started on oral prednisolone 20 mg once a day and a urea cream 40% twice a day, and his pembrolizumab was held. After no major improvement in the rash from the treatment, punch biopsy performed from right forearm lesions confirmed the diagnosis of drug-induced lichenoid dermatitis. The histological features on skin biopsy illustrated basal vacuolization of the dermal-epidermal junction and perifollicular lichenoid inflammatory infiltrations.

## Discussion

The duration and onset of lichenoid drug reactions are often dependent on the causative agent and dosage. The pathogenesis of lichenoid drug reaction is not well-understood. T-cells, keratinocytes, dendritic cells, and endothelial cells, which express activation markers, costimulatory molecules, and adhesion molecules such as microhistocompatibility (MHC) class-II molecules, L-selectin, intercellular adhesion molecule (ICAM) 1 are thought to be involved in the inflammatory reaction that ultimately leads to the apoptosis of basal keratinocyte [3].

Lichenoid drug eruptions are mostly seen in individuals between the age of 57 to 66 years and can have a latent period of one year or more [4]. The clinical presentation (Table 2) and pathological features of lichenoid drug eruptions are similar to lichen planus. Both conditions exhibit erythematous papules and plaques; however, lichenoid drug eruptions may be scaly, highly pruritic, and resolve with greater residual hyperpigmentation [4-5]. Also, Wickham’s striae - a lacy, white network of streaks and the involvement of other mucosal areas are observed less frequently in drug-induced lesions [4-5]. Compared to the flexor surface involvement on extremities with idiopathic lichen planus, lichenoid drug eruption present in a photo-distributed or symmetric pattern [4].

**Table 2:** Lichenoid drug eruption features

Lichenoid drug eruption
Extensive rash: symmetrical over trunks and extremities
Photodistribution: rash in areas exposed to the sun
Rash may be scaly mimicking eczema or psoriasis
Wickam striae usually not seen
Nail and mucus membrane involvement is rare
Pigmentation seen after active rash has cleared

In addition, acanthosis, hypergranulosis, and hyperkeratosis are some of the common characteristic features seen in both lichen planus and lichenoid drug eruptions [6]. However, infiltration of eosinophils in the dermis can delineate lichenoid drug eruption from lichen planus [6].

Lichenoid reactions are chronic inflammatory, T-cell-mediated reactions to an antigen. There are many types of drugs that may trigger this condition (Table 3). In contrast, lichen planus can be associated with systemic conditions such as diabetes mellitus and hepatitis infections.

**Table 3:** Drugs - lichenoid eruption or reaction ACE: angiotensin-converting enzyme; NSAIDs: non-steroidal anti-inflammatory drugs

Anticonvulsants such as carbamazepine or phenytoin
Anti-hypertensives like ACE inhibitors, beta-blockers, nifedipine
Chemotherapy drugs such as fluorouracil, hydroxyurea, or imatinib
Diuretics, like furosemide, hydrochlorthiazide, and spironolactone
HMG-CoA reductase inhibitors
NSAIDs
Hypoglycemic agents
Proton pump inhibitors
Sildenafil citrate
Sulfa drugs, including dapsone, sulfasalazine
Tetracycline
Antitubercular drugs
Tumor necrosis factor antagonists : adalimumab, infliximab
Interferon alfa
Hydroxychloroquine
Misoprostol
Ketoconazole
Phenothiazine derivatives

Lichenoid drug eruptions are usually less likely to resolve and may require discontinuation of the causative agent in addition to topical or oral corticosteroid therapy based on severity. When lichenoid drug eruptions are suspected, the most important step is to discontinue the medication; however, resolution is slow and may take several months to up to 1 year.

Pembrolizumab, a type of immunotherapy, targets PD-1 and has been approved for managing various malignancies viz. melanoma, non-small-cell lung cancer (NSCLC), head and neck squamous cancers, urothelial carcinoma, gastric adenocarcinoma, mismatch-repair-deficient solid tumors, and Hodgkin's lymphoma. Cutaneous toxicities are seen in 30% to 50% of patients treated with various immunotherapies like nivolumab, ipilimumab, and pembrolizumab, etc. [7]. It can manifest as pruritus, vitiligo, lichenoid dermatitis, psoriasiform eruptions, and bullous pemphigoid. Our understanding of cutaneous toxicities stems mostly from ipilimumab use where the overall incidence ranges between the 37% and 70% range for all grades and 1% to 3% for grade 3 or more [8-9]. However, the incidence of grade 3 or higher toxicities of anti-PD-1 agents is the same as with ipilimumab. Although cutaneous toxicities are transient, they can cause significant morbidity and non-compliance with the ongoing treatment regimen, thus leading to impairment of the patient’s health-related quality of life. A similar case of intense lichenoid inflammation and hypergranulosis has been reported in a patient on avelumab, which is also an anti-PD-L1 therapy [10].

The possibility of acute radiation dermatitis at radiation sites causing erythema, hyperpigmentation, pruritus, and burning sensation of skin was also considered in our patient. However, the more conventional, sustained hyperpigmentation or erythema associated with radiotherapy typically does not occur until two to four weeks into treatment [11].

Our case shows that pembrolizumab can induce lichenoid eruption in form of lichenoid dermatitis. It is often characterized by large monomorphic lesions mimicking psoriasiform appearance, with desquamation and crusting that corresponds with the findings observed in our case.

## Conclusions

At present, knowledge of the incidence and diagnosis of various cutaneous irAEs is limited. Discontinuation of immunotherapy remains the only option. As the optimal duration of immunotherapy treatment has not been established in cancer, it is uncertain whether the efficacy of these drugs persists after their discontinuation. Collaboration between oncologists and dermatologists is important in order to understand the spectrum of cutaneous irAEs, optimize their treatment, and define the appropriate timeline of treatment discontinuation, if necessary.
